# Global Pharmacogenomics: Distribution of CYP3A5 Polymorphisms and Phenotypes in the Brazilian Population

**DOI:** 10.1371/journal.pone.0083472

**Published:** 2014-01-10

**Authors:** Guilherme Suarez-Kurtz, Daniela D. Vargens, Ana Beatriz Santoro, Mara H. Hutz, Maria Elisabete de Moraes, Sérgio D. J. Pena, Ândrea Ribeiro-dos-Santos, Marco A. Romano-Silva, Claudio José Struchiner

**Affiliations:** 1 Programa de Farmacologia, Instituto Nacional de Câncer, Rio de Janeiro, RJ, Brazil; 2 Departamento de Genética, Universidade Federal do Rio Grande do Sul, Porto Alegre, RS, Brazil; 3 Departamento de Fisiologia e Farmacologia, Universidade Federal do Ceará, Fortaleza, CE, Brazil; 4 Departamento de Bioquímica e Imunologia, Universidade Federal de Minas Gerais, Belo Horizonte, MG, Brazil; 5 Laboratório de Genética Humana e Médica, Universidade Federal do Pará, Guamá, PA, Brazil; 6 Instituto Nacional de Ciência e Tecnologia de Medicina Molecular, Universidade Federal de Minas Gerais, Belo Horizonte, MG, Brazil; 7 Programa de Computação Científica, Fundação Oswaldo Cruz, Rio de Janeiro, RJ, Brazil; Nanjing Medical University, China

## Abstract

The influence of self-reported “race/color”, geographical origin and genetic ancestry on the distribution of three functional *CYP3A5* polymorphisms, their imputed haplotypes and inferred phenotypes was examined in 909 healthy, adult Brazilians, self-identified as White, Brown or Black (“race/color” categories of the Brazilian census). The cohort was genotyped for CYP3A5*3 (rs776746), CYP3A5*6 (rs10264272) and CYP3A5*7 (rs41303343), *CYP3A5* haplotypes were imputed and CYP3A5 metabolizer phenotypes were inferred according to the number of defective *CYP3A5* alleles. Estimates of the individual proportions of Amerindian, African and European ancestry were available for the entire cohort. Multinomial log-linear regression models were applied to infer the statistical association between the distribution of *CYP3A5* alleles, haplotypes and phenotypes (response variables), and self-reported Color, geographical region and ancestry (explanatory variables). We found that Color *per* se or in combination with geographical region associates significantly with the distribution of *CYP3A5* variant alleles and CYP3A5 metabolizer phenotypes, whereas geographical region *per se* influences the frequency distribution of *CYP3A5* variant alleles. The odds of having the default CYP3A5*3 allele and the poor metabolizer phenotype increases continuously with the increase of European ancestry and decrease of African ancestry. The opposite trend is observed in relation to CYP3A5*6, CYP3A5*7, the default CYP3A5*1 allele, and both the extensive and intermediate phenotypes. No significant effect of Amerindian ancestry on the distribution of *CYP3A5* alleles or phenotypes was observed. In conclusion, this study strongly supports the notion that the intrinsic heterogeneity of the Brazilian population must be acknowledged in the design and interpretation of pharmacogenomic studies, and dealt with as a continuous variable, rather than proportioned in arbitrary categories that do not capture the diversity of the population. The relevance of this work extrapolates the Brazilian borders, and extends to other admixed peoples of the Americas, with ancestral roots in Europe, Africa and the American continent.

## Introduction

*CYP3A5* is one of the four *CYP3A* genes localized in tandem on chromosome 7q21-q22-1, that encode the CYP3A subfamily of enzymes responsible for the metabolism of more than 50% of medicines prescribed worldwide [1]. CYP3A5 is expressed in liver, as well as extra-hepatic tissues such as small intestine, lung, kidney, breast and prostate [2], [3]. The CYP3A5 expression level and enzymatic activity are modulated by genetic polymorphisms. Prominent among these is a 6986A>G transition within intron 3 (rs776746, CYP3A5*3), which leads to an incorrectly spliced mRNA and nonfunctional protein [4]. The worldwide distribution of CYP3A5*3 provides a remarkable example of population diversity, with allele frequencies ranging from 0.14 among sub-Saharan Africans to >0.95 in European populations [5]. In contrast to CYP3A5*3, two other defective *CYP3A5* alleles, namely CYP3A5*6 (rs10264272) - a 14690G>A transition that causes a splice variant mRNA and deletion of exon 7 [4] - and CYP3A5*7 (rs41303343) - a 23132insT mutation that creates a premature stop codon [6] - are relatively frequent in black Africans but are rare or absent in Europeans [5], [7]. European gene inflow in peoples of African ancestry, such as Mixed Ancestry South Africans and African-Americans impacts the distribution of *CYP3A5* polymorphisms and CYP3A5 phenotypes [5], [7]–[9]. In a previous study [10] we examined the influence of African ancestry on the distribution of the CYP3A5*3 allele in residents of Rio de Janeiro, in the Southeast region of Brazil. We now report a comprehensive study of the impact of self-reported Color/race, geographical origin within Brazil and individual proportions of African, European and Amerindian (Native American) ancestry on the distribution of the defective CYP3A5*3, *6 and *7 alleles, inferred *CYP3A5* haplotypes and CYP3A5 metabolizer phenotypes in a large, representative cohort of the present-day Brazilian population.

## Methods

### Ethics Statement

The Ethics Committee of the Instituto Nacional de Câncer (INCA), Rio de Janeiro approved in July 15, 2005 the protocol of the study “Characterizarion of polymorphisms of pharmacogenetic interest and correlation with genetic ancestry” as well as the written Informed Consent form. In August 11, 2008 the same Ethics Committee approved the enlargement of the study and carried forward the approval of the Informed Consent form. The samples were anonymized after collection. Each individual signed a written informed consent.

### Study population

The study cohort consisted of 909 unrelated adults recruited in the North (n = 199), Northeast (214), Southeast (260) and South (236) regions of Brazil. Each individual signed a written informed consent and was asked to self-identify according to the classification scheme adopted by the Brazilian census, which relies on self-perception of skin color [11]. Accordingly, the subjects were distributed in three groups: *branco* (White, n = 308), *pardo* (Brown, n = 296) and *preto* (Black, n = 305). The term Color is capitalized throughout the text to highlight its meaning in the context of the Brazilian census. The study cohort is representative of the overall Brazilian population, since 99% of Brazilians self-identify in one of the three Color categories, and 93% live in one of the four regions, included in the study [12]. All participants have been previously genotyped with a panel of biallelic short insertion-deletion polymorphisms, validated as ancestry informative markers for the Brazilian population [13]. The individual proportions of Amerindian, European and African ancestry were estimated using the *Structure* software [14].

### *CYP3A5* genotyping and inference of haplotypes and phenotypes

Allele discrimination at the CYP3A5*3, *6 and *7 polymorphic loci was performed on a Fast 7500 Real-Time System (Applied Biosystems, Foster City, CA) using TaqMan assays, following the manufacturer's protocols. We denote as CYP3A5*1 all remaining alleles, not detected as CYP3A5*3, CYP3A5*6 or CYP3A5*7. The *CYP3A5* haplotypes comprising the CYP3A5*1 (default),*3, *6 and *7 alleles were imputed using the *Haplo-stats* software [15]. We inferred three CYP3A5 metabolizer phenotypes, according to the number of defective CYP3A5*3, *6 and *7 alleles: individuals having zero, one or two defective alleles were denoted as extensive, intermediate and poor, respectively [16].

The Brazilian census data [12] for the proportion of White, Brown and Black individuals in the four geographical regions included in our study, were used to estimate the “weighted” *CYP3A5* allele, genotype and haplotype frequencies in each Color group and in the overall cohort. To give an example of the procedure employed, White individuals in the North, Northeast, Southeast and South regions represent, respectively, 18.3%, 4.4%, 52.1% and 25.2% of the sum of White individuals in these four regions. By multiplying these percentages by the allele frequency in Whites in the corresponding region, the “weighted” allele frequency for Whites in the overall cohort is obtained.

### Statistical analyses

Allele frequency was derived by gene counting. Deviations from Hardy-Weinberg equilibrium were assessed by the goodness-of-fit χ^2^ test. The allele, haplotype and phenotype frequency data are presented separately for the 12 groups recruited for the study, namely White, Brown and Black individuals in 4 geographical regions. The χ2 or, when appropriate, the Fisher exact test was used to compare the “weighted” allele, genotype and haplotype frequencies among White, Brown and Black Brazilians. We infer the statistical association between the distribution of *CYP3A5* alleles, haplotypes and phenotypes (response variables), and self-reported Color and geographical region (explanatory variables) by fitting multinomial log-linear models via neural networks [17], as described in our previous studies [18], [19]. This procedure obviates the need for correction for multiple comparisons, because the main effects and interaction terms are tested simultaneously within each regression context. In multinomial log-linear modeling of the association between biogeographical ancestry and distribution of *CYP3A5* alleles and inferred phenotypes in Brazilians, ancestry entered the model transformed as a piecewise polynomial, and the result of the fitting exercise is presented in graphic format relating the proportion of individuals with the variant marker against the proportion of ancestry in a cluster specified by the *Structure* software [14]. This method is implemented as function ‘multinom’ available in the R package and package ‘splines’ [20]. B-splines were fit with parameter ‘degree = 3’ (cubic b-splines). Function “Anova” available in the “car” package under R calculates type-II or type-III analysis-of-variance tables for model objects produced by multinom [21].

The level of significance of all statistical analyses was set at *P*<0.05.

## Results

Table 1 displays the frequency distribution of *CYP3A5* alleles, imputed haplotypes and inferred CYP3A5 metabolizer phenotypes among Brazilians, stratified by geographical region and self-reported Color. Genotype frequency at each polymorphic locus did not deviate significantly from Hardy-Weinberg expectations in the overall study cohort. Color *per* se or in combination with geographical region was significantly associated with the frequency distribution of the *CYP3A5* variant alleles and CYP3A5 metabolizer phenotypes, whereas geographical region *per se* associated with the frequency distribution of *CYP3A5* variant alleles, but not CYP3A5 phenotypes (Table 2).

**Table 1:** Distribution of *CYP3A5* alleles and haplotypes, and CYP3A5 phenotypes among Brazilians, according to geographical region and self-reported Color.

Region/Color	Northeast			North			Southeast			South		
	White (n = 75)	Brown (n = 67)	Black (n = 72)	White (n = 64)	Brown (n = 63)	Black (n = 72)	White (n = 88)	Brown (n = 87)	Black (n = 85)	White (n = 81)	Brown (n = 79)	Black (n = 76)
**Alleles**												
*1	0.24	0.22	0.31	0.20	0.25	0.36	0.13	0.27	0.33	0.14	0.35	0.35
*3	0.72	0.69	0.53	0.79	0.72	0.56	0.84	0.64	0.45	0.85	0.55	0.49
*6	0.03	0.05	0.10	0.01	0.02	0.05	0.02	0.06	0.12	0.00	0.04	0.11
*7	0.01	0.02	0.06	0.00	0.00	0.03	0.02	0.03	0.10	0.01	0.06	0.07
**Haplotypes**												
*1/*1	0.04	0.03	0.13	0.08	0.05	0.10	0.00	0.08	0.12	0.00	0.15	0.13
*1/*3	0.33	0.33	0.28	0.25	0.40	0.44	0.23	0.30	0.26	0.28	0.35	0.26
*1/*6	0.01	0.04	0.06	0.00	0.02	0.04	0.01	0.05	0.07	0.00	0.03	0.09
*1/*7	0.01	0.03	0.03	0.00	0.00	0.04	0.01	0.03	0.09	0.00	0.01	0.05
*3/*3	0.52	0.51	0.29	0.66	0.51	0.31	0.70	0.45	0.24	0.70	0.29	0.29
*3/*6	0.01	0.03	0.10	0.02	0.03	0.06	0.02	0.07	0.11	0.00	0.05	0.08
*3/*7	0.01	0.01	0.10	0.00	0.00	0.01	0.02	0.02	0.06	0.01	0.11	0.08
*6/*6	0.01	0.01	0.03	0.00	0.00	0.00	0.00	0.00	0.01	0.00	0.00	0.03
*6/*7	0.00	0.00	0.00	0.00	0.00	0.00	0.00	0.00	0.05	0.00	0.00	0.00
**Phenotypes**												
Extensive	0.04	0.03	0.13	0.08	0.05	0.10	0.00	0.08	0.12	0.00	0.15	0.13
Intermediate	0.40	0.40	0.36	0.25	0.41	0.53	0.25	0.38	0.42	0.28	0.39	0.39
Poor	0.56	0.57	0.51	0.67	0.54	0.38	0.75	0.54	0.46	0.72	0.46	0.47

n = number of individuals.

**Table 2:** Multinomial log-linear analyses of the distribution of *CYP3A5* alleles and CYP3A5 phenotypes among Brazilians according to self-reported Color and geographical region.

	Explanatory variables^a^		
	Color	Geographical region	Color∶geographical region
Allele	<0.0001	0.001	0.037
Phenotype	<0.0001	0.796	0.002

^a^ *p* values associated to the “main effects” (Color and geographical region) and their “interaction”.

Table 3 presents the weighted frequency of *CYP3A5* alleles, haplotypes and phenotypes in the White, Brown and Black Brazilian population and the results of statistical analyses of these data. Highly significant differences (*P*<0.0001) were observed in allele, haplotype, and phenotype distribution across the three Color groups.

**Table 3:** Frequency of *CYP3A5* alleles and haplotypes, and CYP3A5 phenotypes in Brazilians.

*CYP3A5*	Overall cohort (909)	Self-identified Color groups			
		White (308)	Brown (296)	Black (305)	*P* value^a^
**Alleles**					
*1	0.21	0.15	0.26	0.32	<0.0001
*3	0.73	0.82	0.68	0.52	
*6	0.04	0.02	0.05	0.11	
*7	0.03	0.01	0.04	0.08	
**Haplotypes**					
*1/*1	0.04	0.01	0.06	0.12	<0.0001
*1/*3	0.29	0.27	0.32	0.28	
*1/*6	0.03	0.01	0.04	0.06	
*1/*7	0.02	0.01	0.03	0.06	
*3/*3	0.55	0.67	0.47	0.27	
*3/*6	0.03	0.02	0.05	0.10	
*3/*7	0.04	0.01	0.06	0.12	
*6/*6	0.01	0	0.01	0.02	
*6/*7	0	0	0	0.02	
**Phenotypes**					
Full-activity	0.04	0.01	0.06	0.12	<0.0001
Reduced-activity	0.34	0.29	0.39	0.41	
Null-activity	0.62	0.7	0.55	0.47	

a Comparison across the three Color groups (chi square or Fisher exact tests).

**Figure 1:**
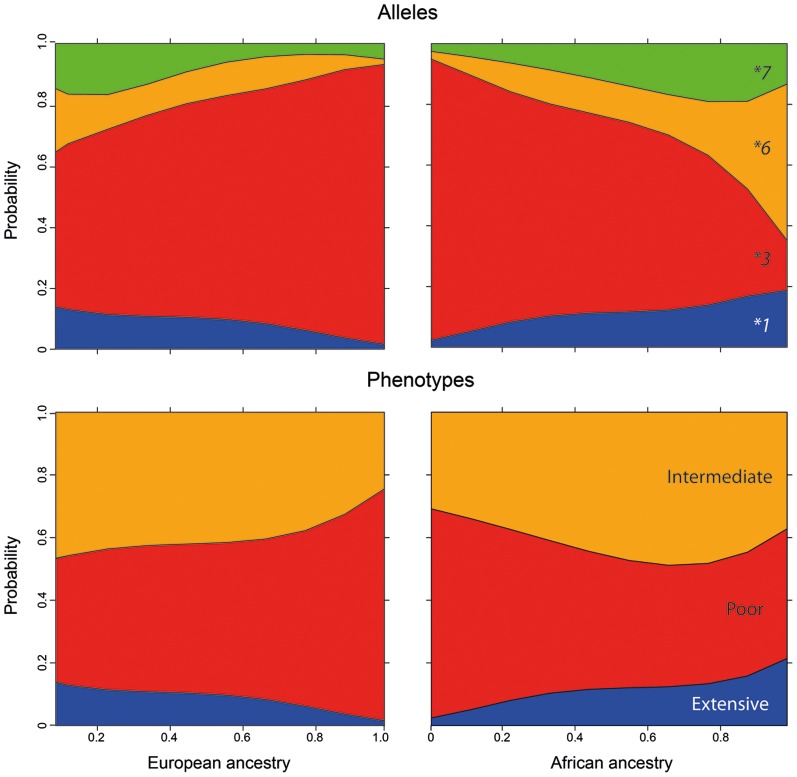
Fitted logistic models showing the probability proportions describing the association between European ancestry and distribution frequency of *CYP3A5* alleles (A) and CYP3A5 metabolizer phenotypes (B) in 909 healthy Brazilians.

Figure 1 presents the best fitting models for the association between the individual proportions of biogeographical ancestry and frequency distribution of *CYP3A5* alleles and inferred CYP3A5 phenotypes. It is apparent that the odds of having the defective CYP3A5*3 allele increase continuously as European ancestry increases, and African ancestry decreases in the overall cohort. The opposite trend is observed in relation to CYP3A5*6, CYP3A5*7, and the default CYP3A5*1 allele, all of which increase in frequency as African ancestry increases and European ancestry decreases. Regarding CYP3A5 phenotypes, the best fitting models show increasing odds of having the poor metabolizer phenotype as European ancestry increases and African ancestry decreases, whereas the opposite trend prevails for the extensive and intermediate phenotypes. No significant effect of Amerindian ancestry on the distribution of *CYP3A5* alleles or phenotypes was observed, which may be explained by the relatively small average proportion (<10%) of Amerindian ancestry in the overall Brazilian population, compared to European and African ancestry [22], [23].

## Discussion

The present-day Brazilian population, in excess of 195 million people, is extensively admixed, with major ancestral roots in Europe, Africa and America. The extent of admixture, predominantly between European and African ancestors, is reflected in the large percentage (43.1%) of Brazilians who self-identify as “Pardo” (meaning Brown) in the Brazilian census [12]. For comparison, 47.8% and 8.2% of the population self-reported as White or Black, respectively, whereas the two other Color/race categories of the census, namely Amerindian (Native Americans) and Yellow (meaning of Asian extraction) accounted for less than 1% of the overall Brazilian population. Not surprisingly, in a country of continental dimensions (8.5 million km^2^), with uneven distribution of Native peoples and five centuries of distinct immigration patterns, the extent and characteristics of admixture vary largely across geographical regions, as well as within the Color/race categories adopted by the Brazilian census. As a consequence, at the individual level there is significant dissociation of Color and biogeographical ancestry in Brazilians, and the proportions of European and African ancestry vary continuously, irrespective of Color/race categories [22], [23]. We have previously shown that this pattern is mirrored in the distribution of several pharmacogenetic polymorphisms [10], [18], [19], [23], and now extend this observation to *CYP3A5* polymorphisms and metabolizer phenotypes.

One distinct feature of the present study is the application of multinomial non-linear regression modeling to data from a large, representative cohort of the overall Brazilian population. This approach allowed us to demonstrate that the frequency distribution of the CYP3A5*3, *6 and *7 alleles, their inferred haplotypes and phenotypes varies significantly according to self-reported Color and geographical origin within Brazil. The influence of Color remained significant after adjustment (“weighting”) of the allele, haplotype and phenotype frequencies according to the most recent Brazilian census data. These findings are in line with our previous results for *VKORC1*, *ABCB1* and the *CYP2C* cluster genes [19], [20], [24], and collectively, represent a caveat against extrapolation of pharmacogenomic data from cohorts recruited at one or a few study sites to the overall Brazilian population. This concern is especially relevant in the case of polymorphisms, such as CYP3A5*3, *6 and *7, which occur at markedly different frequencies in Europeans and sub-Saharan Africans, the two major ancestral roots of Brazilians (Introduction) [2].

Modeling the impact of biogeographical ancestry on the distribution of *CYP3A5* polymorphisms, revealed that the odds of having the CYP3A5 *1, *3 *6 or *7 alleles, and the extensive, intermediate or poor CYP3A5 phenotypes vary continuously across the study cohort, according to the individual proportions of European or African ancestry. The increasing odds of having the CYP3A5*3 allele as the individual proportion of European ancestry increases and African ancestry declines, observed throughout the study cohort, irrespective of self-reported Color, is consistent with the much higher frequency of CYP3A5*3 in Europeans compared to Africans (see Introduction). By the same token, the opposite trend in the distribution of the CYP3A5*6 and *7 alleles, is readily accounted by their occurrence at frequencies that may exceed 0.2 in black Africans, while being absent or extremely rare in Europeans [5], [7], [25]. Because the CYP3A5 phenotypes were inferred from the individual *CYP3A5* haplotypes, the best fit regression models for the association between biogeographical ancestry and CYP3A5 phenotype distribution among Brazilians reflect the differential distribution of *CYP3A5* polymorphisms in European *versus* black African peoples.

The present study confirms, and extends in several directions our previous report of the influence of the African component of ancestry on the distribution of CYP3A5*3 in an admixed cohort from Rio de Janeiro [10]. First, we included in our analyses two other functional polymorphisms, namely CYP3A5*6 and CYP3A5*7, haplotypes comprising the CYP3A5*1 (default), *3, *6 and *7* alleles, and the inferred CYP3A5 metabolizer phenotypes. Second, we enrolled individuals from three other geographical regions of Brazil, which combined with the Southeast region represent 93% of the country's population. Third, we examined separately the influence of European, African and Amerindian biogeographical ancestry on the distribution of *CYP3A5* polymorphisms and metabolizer phenotypes in Brazilians. The findings in both the present and the previous study [10] provide strongly support the notion that the intrinsic heterogeneity of the Brazilian population must be acknowledged in the design and interpretation of pharmacogenomic studies, and dealt with as a continuous variable, rather than proportioned in arbitrary categories that do not capture the diversity of the population. The relevance of this work extrapolates the Brazilian borders, and extends to other admixed peoples of the Americas, with ancestral roots in Europe, Africa and the American continent [26], [27].
